# Investigations in the possibility of early detection of colorectal cancer by gas chromatography/triple-quadrupole mass spectrometry

**DOI:** 10.18632/oncotarget.15081

**Published:** 2017-02-04

**Authors:** Shin Nishiumi, Takashi Kobayashi, Shuichi Kawana, Yumi Unno, Takero Sakai, Koji Okamoto, Yasuhide Yamada, Kazuki Sudo, Taiki Yamaji, Yutaka Saito, Yukihide Kanemitsu, Natsuko Tsuda Okita, Hiroshi Saito, Shoichiro Tsugane, Takeshi Azuma, Noriyuki Ojima, Masaru Yoshida

**Affiliations:** ^1^ Division of Gastroenterology, Department of Internal Medicine, Kobe University Graduate School of Medicine, Chuo-ku, Kobe, Hyogo 650-0017, Japan; ^2^ Analytical and Measuring Instruments Division, Shimadzu Corporation, Nakagyo-ku, Kyoto 604-8511, Japan; ^3^ Division of Cancer Differentiation, National Cancer Center Research Institute, Tokyo 104-0045, Japan; ^4^ Department of Gastrointestinal Medical Oncology, National Cancer Center Hospital, Chuo-ku, Tokyo 104-0045, Japan; ^5^ Division of Epidemiology, Center for Public Health Sciences, National Cancer Center, Chuo-ku, Tokyo 104-0045, Japan; ^6^ Endoscopy Division, National Cancer Center Hospital, Chuo-ku, Tokyo 104-0045, Japan; ^7^ Department of Colorectal Surgery, National Cancer Center Hospital, Chuo-ku, Tokyo 104-0045, Japan; ^8^ Division of Screening Assessment and Management, Center for Public Health Sciences, National Cancer Center, Chuo-ku, Tokyo 104-0045, Japan; ^9^ Center for Public Health Sciences, National Cancer Center, Chuo-ku, Tokyo 104-0045, Japan; ^10^ Division of Metabolomics Research, Department of Internal Related, Kobe University Graduate School of Medicine, Chuo-ku, Kobe, Hyogo 650-0017, Japan; ^11^ AMED-CREST, AMED, Chuo-ku, Kobe, Hyogo 650-0017, Japan

**Keywords:** metabolomics, colorectal cancer, biomarker, gas chromatography/triple-quadrupole mass spectrometry, metabolite

## Abstract

In developed countries, the number of patients with colorectal cancer has been increasing, and colorectal cancer is one of the most common causes of cancer death. To improve the quality of life of colorectal cancer patients, it is necessary to establish novel screening methods that would allow early detection of colorectal cancer. We performed metabolome analysis of a plasma sample set from 282 stage 0/I/II colorectal cancer patients and 291 healthy volunteers using gas chromatography/triple-quadrupole mass spectrometry in an attempt to identify metabolite biomarkers of stage 0/I/II colorectal cancer. The colorectal cancer patients included patients with stage 0 (N=79), I (N=80), and II (N=123) in whom invasion and metastasis were absent. Our analytical system detected 64 metabolites in the plasma samples, and the levels of 29 metabolites differed significantly (Bonferroni-corrected p=0.000781) between the patients and healthy volunteers. Based on these results, a multiple logistic regression analysis of various metabolite biomarkers was carried out, and a stage 0/I/II colorectal cancer prediction model was established. The area under the curve, sensitivity, and specificity values of this model for detecting stage 0/I/II colorectal cancer were 0.996, 99.3%, and 93.8%, respectively. The model's sensitivity and specificity values for each disease stage were >90%, and surprisingly, its sensitivity for stage 0, specificity for stage 0, and sensitivity for stage II disease were all 100%. Our predictive model can aid early detection of colorectal cancer and has potential as a novel screening test for cases of colorectal cancer that do not involve lymph node or distant metastasis.

## INTRODUCTION

In developed countries, colorectal cancer is one of the most common causes of cancer death [1], and it is treated using a combination of colonoscopy, surgery, chemotherapy, and radiotherapy. When colorectal cancer is discovered early, its 5-year relative survival rate is very high, but advanced colorectal cancer reduces the quality of life (QOL) of patients. Therefore, novel methods that would allow the early detection and diagnosis of colorectal cancer are desired in the medical field. The fecal occult blood test (FOBT) and blood tests for tumor markers are commonly used as screening methods for diagnosing colorectal cancer. The FOBT is a non-invasive and inexpensive method, but has low sensitivity and specificity for cases of colorectal cancer that do not involve lymph node or distant metastasis. As for blood tests for tumor markers, carcinoembryonic antigen (CEA) and carbohydrate antigen 19-9 (CA19-9) can be used to test for colorectal cancer, but such blood tests also have low sensitivity so they are not appropriate for detecting colorectal cancer early. Colonoscopy is a more accurate and reliable method for detecting colorectal cancer early, but it cannot be used for screening because it is more invasive and expensive than blood testing. Recently, computed tomographic colonography, which is less invasive than colonoscopy, has been used to detect colorectal cancer; however, special X-ray equipment is required to obtain detailed scans, which is expensive. Combinations of conventional screening methods are also used to detect colorectal cancer, but such combined approaches only detect about 40% of colorectal cancer cases [2].Therefore, it is necessary to establish the novel screening methods for early detection of colorectal cancer with high sensitivity, high specificity, noninvasive and easy procedure.

Biomarker research into colorectal cancer is being carried out using a variety of approaches including omics-based methods, such as genomics and proteomics, and many researchers are searching for novel biomarkers that would allow the early detection of the disease and could also be used for predicting therapeutic efficacy, recurrence, and prognosis, etc. [3]. In our previous study, serum metabolomics or metabolome analysis, which is the comprehensive study of low molecular weight metabolites, was employed to find novel metabolite biomarkers of colorectal cancer, and then a colorectal cancer prediction model based on four metabolites was established using multiple logistic regression analysis [4]. The metabolome mainly represents the endpoint of the omics cascade, and it is also the closest point in the cascade to the phenotype. The genome, which is located in the upstream part of the omics cascade and includes numerous genes, is generally not affected by exogenous factors, such as environmental and dietary factors. Even if a certain gene contains a mutation, it might not affect the rest of the body due to the effects of homeostatic functions. In addition to variations in DNA, mRNA, and protein expression, the metabolome is affected by the enzymatic activities of various proteins, and alterations in the levels of metabolites can also be caused by exogenous factors. Therefore, the metabolome could be considered to be a summary of the other upstream omics profiles, and metabolome analysis might be able to detect subtle changes in metabolic pathways and deviations from homeostasis before phenotypic changes occur [5, 6]. Thus, metabolomics could contribute greatly to biomarker research [7, 8]. Our previous study [4], in which a colorectal cancer prediction model was established, included colorectal cancer patients with stage 0 to IV disease, and the colorectal cancer prediction model demonstrated sensitivity and specificity values of about 80%. However, the number of stage 0/I/II colorectal cancer patients was relatively small, so it is necessary to carry out a further trial involving a larger number of stage 0/I/II colorectal cancer patients in order to establish a screening procedure that could contribute to the early detection of colorectal cancer and improve the QOL of colorectal cancer patients.

In this study, we employed gas chromatography/triple-quadrupole mass spectrometry (GC/QqQMS), which is generally used for multiple reaction monitoring (MRM) analysis. QqQMS-based MRM analysis can be used to easily distinguish single metabolite-derived peaks from co-eluted peaks and background noise, and it exhibits high sensitivity and a wide dynamic range compared with single-QMS [9], although gas chromatography/single-quadrupole mass spectrometry (GC/QMS) is very useful for comprehensive metabolite analysis [10]. In our previous study [4], GC/QMS was used to discover biomarker candidates for colorectal cancer. An automatic derivatization machine was also used because inter-sample differences in the time between derivatization and measurement can affect the results [11, 12]. By using this automatic derivatization machine, our GC/QqQMS analysis system is able to obtain more accurate metabolite data. The aim of the present study is to find metabolite biomarker candidates that would allow the detection of cases of colorectal cancer that do not involve lymph node or distant metastasis. To do this, human plasma samples that were collected from stage 0 to stage II colorectal cancer patients and corresponding healthy volunteers were analyzed using our GC/QqQMS analysis system. Then, a multiple logistic regression model for detecting cases of colorectal cancer without any lymph node or distant metastasis was established on the basis of the metabolite data.

## RESULTS

Our GC/QqQMS-based metabolite analysis system detected 64 metabolites in the subjects’ plasma. The sample set examined in this study was obtained from 282 colorectal cancer patients and 291 healthy volunteers, and the colorectal cancer patients included clinical stage 0 (N=79), I (N=80), and II (N=123) patients who were free from invasion and metastasis. Regarding the characteristics of the colorectal cancer patients and healthy volunteers included in this study (Table 1), there were no significant differences in age or body mass index (BMI) between the colorectal cancer patients and healthy volunteers. The male-to-female ratio of the two groups also did not differ significantly. Significant differences in the frequencies of current smokers, former smokers, and people who had never smoked were observed between the colorectal cancer patients and healthy volunteers, whereas the significance of the inter-group differences in the frequencies of current alcohol drinkers, former alcohol drinkers, and people who had never drank alcohol could not be confirmed. The numbers of people who were and were not taking medication differed significantly between the colorectal cancer patients and healthy volunteers. As for the blood test results, the blood levels of total cholesterol, triglycerides, glucose, total bilirubin, aspartate transaminase (AST), C-reactive protein (CRP), CEA, and CA19-9 differed significantly between the colorectal cancer patients and healthy volunteers (Supplementary Table 1).

**Table 1:** Characteristics of the colorectal cancer patients and healthy volunteers

		CRC	HV	p-value
	N	282	291	
Age (y.o.)	Median	68 (40-93)	68 (41-88)	0.825
	Mean	67.0	66.8	
	S.D.	9.02	7.94	
				
Sex	Male	170	178	0.828
	Female	112	113	
BMI (kg/m^2^)	Mean	22.9	22.8	0.6119
	S.D.	3.62	2.82	
				
Smoking habits	Current	52	24	0.0006
	Former	116	118	
	Never	114	149	
				
Alcohol consumption	Current	174	198	0.1132
	Former	26	18	
	Never	79	75	
	N.R.	3	0	
				
Medication	Yes	160	202	0.0017
	No	122	89	
				
Tumor (T)	Tis	79		
	T1	53		
	T2	27		
	T3	119		
	T4a	2		
	T4b	2		
				
Lymph node (N)	N0	282		
	N1/2	0		
				
Metastasis (M)	M0	282		
	M1	0		
				
Stage	0	79		
	I	80		
	II	123		
				
Site	C	33		
	A	39		
	T	23		
	D	11		
	S	73		
	R	101		
	P	2		
				
Histology	tub1	175		
	tub2	91		
	por1	2		
	por2	1		
	pap	3		
	muc	10		

The p-values for age and BMI were calculated using Welch's t-test. The p-values for sex, smoking habits, alcohol consumption, medication usage, and cancer stage were calculated using Pearson's chi-squared test. CRC: colorectal cancer patients; HV: healthy volunteers; y.o.: years old; BMI: body mass index; S.D.: standard deviation; N.R.: no response; C: cecum; A: ascending colon; T: transverse colon; D: descending colon; S: sigmoid colon; R: rectum; P: sacral promontory.

The plasma metabolite levels of the colorectal cancer patients and healthy volunteers were compared using Wilcoxon's rank sum test (Supplementary Table 2). As a result, the plasma levels of 41 of the 64 metabolites were shown to differ significantly (p<0.05) between the colorectal cancer patients and healthy volunteers. After the application of Bonferroni's correction (p=0.000781), the levels of 29 metabolites continued to exhibit significant differences. Next, a simple linear regression analysis of the 29 metabolites that demonstrated Bonferroni-corrected significant differences was performed, and their area under the curve (AUC), sensitivity, and specificity values were evaluated (Table 2). Pyruvic acid-meto-trimethylsilyl (TMS), glycolic acid-2TMS, lactic acid-2TMS(/SI), and fumaric acid-2TMS(/SI) displayed AUC values of >0.8, and both the sensitivity and specificity of pyruvic acid-meto-TMS and lactic acid-2TMS(/SI) were >80%.

**Table 2:** AUC, sensitivity, and specificity values of the metabolites that exhibited Bonferroni-corrected significant differences

	AUC	Sensitivity	Specificity
Pyruvic acid-meto-TMS	0.93551	87.9%	90.4%
Glycolic acid-2TMS	0.90352	92.6%	78.0%
Lactic acid-2TMS(/SI)	0.88963	82.6%	82.8%
Fumaric acid-2TMS(/SI)	0.83445	74.1%	80.8%
Ornithine-4TMS(/SI)	0.76751	68.8%	76.3%
Sucrose-8TMS	0.7613	74.8%	66.0%
Fructose-meto-5TMS(2)	0.71667	71.6%	65.3%
Arabinose-meto-4TMS	0.71089	65.3%	69.1%
2-ketoglutaric acid-meto-2TMS	0.67883	64.9%	66.3%
Sorbose-meto-5TMS(1)	0.66413	67.7%	61.9%
Palmitoleic acid-TMS	0.65756	55.3%	69.4%
Tryptophan-3TM(/SI)	0.6573	45.0%	81.1%
Cysteine-3TMS	0.64901	79.1%	46.4%
Xylose-meto-4TMS(2)	0.6352	51.4%	71.1%
2-aminobutyric acid-2TMS	0.62659	72.7%	50.5%
Lysine-4TMS	0.62194	39.0%	81.8%
Malic acid-3TMS(/SI)	0.62077	42.2%	77.0%
Threitol-4TMS	0.62045	39.0%	80.4%
Maltose-meto-8TMS(1)	0.38827	12.4%	96.2%
Elaidic acid-TMS	0.6098	53.9%	67.0%
Uric acid-4TMS	0.60658	51.1%	69.4%
Isocitric acid-4TMS	0.60463	35.5%	85.6%
meso-erythritol-4TMS	0.60291	42.9%	79.0%
Valine-2TMS(/SI)	0.595	47.2%	69.4%
Leucine-2TMS	0.59334	49.3%	65.6%
3-hydroxyisovaleric acid-2TMS	0.59258	38.3%	78.7%
Xylitol-5TMS	0.5882	62.8%	56.4%
Arabitol-5TMS	0.58759	57.1%	60.8%
Proline-2TMS	0.58217	54.6%	61.9%

A simple linear regression analysis of the metabolites that exhibited Bonferroni-corrected significant differences was performed, and the AUC, sensitivity, and specificity values of these metabolites are shown in Table 2. AUC: area under the curve; TMS: trimethylsilyl group; SI: stable isotope; ‘-TMS’: the number of TMS molecules bound to each metabolite via derivatization; ‘/SI’: the metabolites whose peak intensity values were normalized using the corresponding stable isotopes.

**Figure 1:**
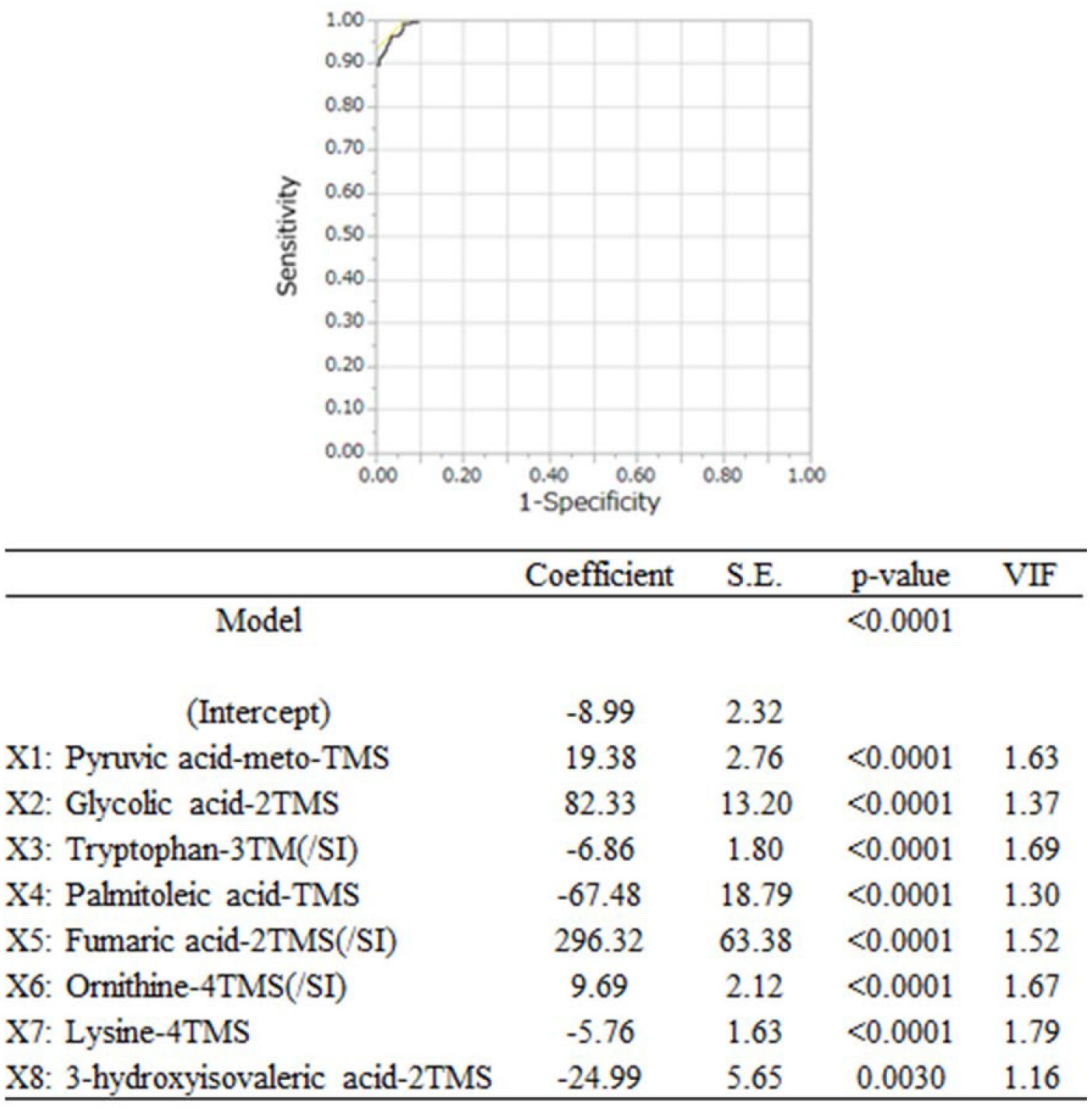
The ROC curve and data for the predictive model The black line on the graph is the ROC curve for the predictive model. The AUC, sensitivity, and specificity values of the predictive model obtained via multiple logistic regression analysis were 0.996, 99.3%, and 93.8%, respectively, and the optimal cut-off value was 0.19. The coefficients, S.E., p-values, and VIF of this predictive model at the intercept and for each variable are shown in the table below the graph. S.E.: standard error; VIF: variance inflation factor; TMS: trimethylsilyl group; SI: stable isotope; ‘-TMS’: the number of TMS molecules bound to each metabolite via derivatization; ‘/SI’: the metabolites whose peak intensity values were normalized using the corresponding stable isotopes.

Based on these results, multiple logistic regression analysis involving various metabolite biomarkers was performed. The 29 metabolites that exhibited Bonferroni-corrected significant inter-group differences were subjected to a stepwise variable selection method, and 8 metabolites (pyruvic acid-meto-TMS, glycolic acid-2TMS, tryptophan-3TMS(/SI), palmitoleic acid-TMS, fumaric acid-2TMS(/SI), ornithine-4TMS(/SI), lysine-4TMS, and 3-hydroxyisovaleric acid-2TMS) were selected as variables for the multiple logistic regression model. Then, a multiple logistic regression model composed of these 8 selected metabolites was established, which resulted in the following predictive model (Figure 1):

p=1/[1+exp^-{-8.99+19.38×1+82.33×2-6.86×3-67.48×4+296.32×5+9.69×6-5.76×7-24.99×8}^]

The AUC, sensitivity, and specificity values of this model were 0.996, 99.3%, and 93.8%, respectively (Table 3). On the other hand, the sensitivity of CEA and CA19-9 were both <20%, although the specificity of CEA and CA19-9 were both >95% (Table 3). The sensitivity/specificity of the developed model for each clinical stage (0/I/II) were also evaluated (Table 3). As a result, it was found that the model exhibited sensitivity and specificity values of >90% for each stage of the disease, and surprisingly its sensitivity for stage 0 disease, specificity for stage 0 disease, and sensitivity for stage II disease were all 100%. On the contrary, the sensitivity of CEA was 3.8% for stage 0 disease, 15.0% for stage I disease, and 29.3% for stage II disease, and the sensitivity of CA19-9 was 6.4% for stage 0 disease, 6.3% for stage I disease, and 13.1% for stage II disease. CEA and CA19-9 exhibited specificity values of >90% for each stage of the disease.

**Table 3:** Sensitivity and specificity of the predictive model and tumor markers

		Stage 0/I/II	Stage 0	Stage I	Stage II
Sensitivity	Model	99.3%	100%	97.5%	100%
	CEA	18.1%	3.8%	15.0%	29.3%
	CA19-9	9.3%	6.4%	6.3%	13.1%
					
Specificity	Model	93.8%	100%	91.3%	91.7%
	CEA	96.0%	92.4%	97.4%	97%
	CA19-9	95.6%	95.5%	97.4%	94.4%

The sensitivity and specificity values of the predictive model obtained via multiple logistic regression analysis, and CEA and CA19-9, which were selected via simple linear regression analysis, are shown. Sensitivity and specificity were separately evaluated for stage 0-II (0/I/II), stage 0, stage I, and stage II disease. CEA: carcinoembryonic antigen; CA19-9: carbohydrate antigen 19-9.

Finally, the AUC of the predictive model was compared with that of pyruvic acid-meto-TMS (Supplementary Figure 1) because the AUC of pyruvic acid-meto-TMS was especially high in the simple linear regression analysis. As a result, the AUC of the predictive model was shown to be significantly superior to that of pyruvic acid-meto-TMS (p<0.0001).

## DISCUSSION

In this study, we investigated whether the alterations in plasma metabolite levels are available for early detection of colorectal cancer. An analytical system composed of an automatic derivatization machine and GC/QqQMS was used for this investigation. The automatic derivatization machine helped to eliminate the influence of inter-sample differences in the time between derivatization and measurement on plasma metabolite levels. In addition, GC/QqQMS makes it possible to easily distinguish the peak for a particular metabolite from co-eluted peaks and background noise, resulting in higher quality evaluations. The aim of this study is to find metabolite biomarkers and/or multiple metabolite-based multiple logistic regression models that would allow the discovery and diagnosis of cases of colorectal cancer that do not involve lymph node or distant metastasis. Therefore, the sample set was composed of colorectal cancer patients with stage 0, I, or II disease and corresponding healthy volunteers.

Some previous metabolomics studies have attempted to discover biomarkers that might aid the early detection of colorectal cancer. Ritchie et al. performed non-targeted and targeted serum metabolite profiling using training sets composed of three and two independent cohorts, respectively, and 28-36 carbon-containing hydroxylated polyunsaturated ultra-long-chain fatty-acids were identified as biomarkers of colorectal cancer [13]. In this study by Ritchie et al., 13 to 34 stage 0/I/II colorectal cancer patients were included in each cohort, and 28-36 carbon-containing hydroxylated polyunsaturated ultra-long-chain fatty acids were shown to be useful for discovering stage 0/I/II colorectal cancer patients. In a study by Miyagi et al., two-class linear discrimination analysis based on plasma amino acid profiling showed AUC values of 0.903, 0.859, and 0.829 for detecting stage 0 (n=8), I (n=63), and II (n=48) colorectal cancer, respectively [14]. A study by Fang et al., which included 26 stage I/II colorectal cancer patients, found that the serum levels of some lipid species differed significantly between stage I/II colorectal cancer patients and healthy volunteers [15]. Proton nuclear magnetic resonance spectroscopy (^1^HNMR)-based fecal metabolomic fingerprinting was reported to be useful for distinguishing stage I/II colorectal cancer patients from healthy volunteers in a study involving 20 stage I/II colorectal cancer patients [16]. In a study by Uchiyama et al. based on capillary electrophoresis/mass spectrometry [17], in which 14 stage I colorectal cancer patients and 14 stage II colorectal cancer patients were included in the sample set, the serum level of benzoic acid showed sensitivity, specificity, and AUC values of 1.0, 0.98, and 0.98, respectively, for stage I colorectal cancer, and 0.93, 0.90, and 0.95, respectively, for stage II colorectal cancer. Therefore, previous studies have identified promising metabolite biomarkers of cases of colorectal cancer that do not involve lymph node or distant metastasis, but many of these studies included small numbers of colorectal cancer patients that were free from lymph node and distant metastasis; i.e., stage 0/I/II colorectal cancer patients. Our study solely involved patients with stage 0 (n=79), I (n=80), or II (n=123) colorectal cancer. Moreover, the established predictive model exhibited sensitivity of >95%, and its specificity was also >90% (Table 3). These results suggest that the predictive model established in our study may be superior to other biomarker candidates reported in previous studies.

The established predictive model was composed of 8 metabolites; i.e., pyruvic acid, glycolic acid, tryptophan, palmitoleic acid, fumaric acid, ornithine, lysine, and 3-hydroxyisovaleric acid. The multicollinearity of the metabolites could not be confirmed because their variance inflation factor (VIF) values were small (Figure 1). Therefore, a variety of the factors associated with colorectal cancer without any lymph node or distant metastasis might have independently contributed to the alterations in the blood levels of each metabolite. For example, the colorectal cancer patients had higher plasma levels of pyruvic acid, which is an intermediate of glycolysis, than the healthy volunteers (Supplementary Table 2). The colorectal cancer patients also had higher plasma levels of lactic acid, which is the end product of glycolysis, than the healthy volunteers (Supplementary Table 2). A previous proteomic analysis of colorectal cancer tissues obtained from stage III colorectal cancer patients revealed the upregulation of glycolysis [18]. On the other hand, Hirayama et al. found that colorectal cancer tissue contained lower levels of pyruvic acid than normal colon tissue, although the concentration of lactic acid was increased in the colorectal cancer tissue [19]. This phenomenon is known as ‘the Warburg effect’. The colorectal cancer tissue examined in the study by Hirayama et al. was collected from colorectal cancer patients with stage I to IV disease. The blood level of pyruvic acid also reflects the nutritional state of the body regardless of the presence/absence of cancer. The plasma level of fumaric acid, which is an intermediate of the tricarboxylic acid (TCA) cycle (which occurs downstream of glycolysis), was also found to be increased in colorectal cancer patients compared with healthy volunteers, and moreover, the levels of 2-ketoglutaric acid, malic acid, and isocitric acid, which are intermediates of the TCA cycle, were significantly higher in the colorectal cancer patients (Supplementary Table 2). Glycolysis and the TCA cycle are important metabolic pathways for providing energy to cells, and the abovementioned findings indicate that information about local colorectal cancer-based alterations in nutritional conditions should be included in predictive models for colorectal cancer.

Palmitoleic acid, which is a ω-7 unsaturated fatty acid, is abundant in dietary oils, and it is also produced from palmitic acid by Δ9 desaturase in the body. In the present study, the colorectal cancer patients displayed significantly lower levels of palmitoleic acid than the healthy volunteers (Supplementary Table 2). The colorectal cancer patients exhibited lower blood levels of total cholesterol than the healthy volunteers, but their blood levels of triglycerides were higher than those of the colorectal cancer patients (Supplementary Table 1), suggesting that there is no relationship between stage 0/I/II colorectal cancer and lipid metabolism abnormalities. Palmitoleic acid enhances insulin sensitivity by suppressing inflammation and inhibiting the destruction of insulin-secreting pancreatic β cells [20], and it also reduces obesity-related inflammation [21]. Thus, palmitoleic acid seems to exert anti-inflammatory effects, and its administration to humans was shown to decrease blood CRP levels [22]. It has also been suggested that changes in a person's fatty acid profile might affect their risk of colorectal cancer. [23]. Therefore, a reduced level of palmitoleic acid could lead to altered inflammatory conditions in the body, and hence, influence the development of stage 0/I/II colorectal cancer. Accordingly, it might be beneficial to include information about inflammatory conditions in predictive models for colorectal cancer.

In this study, a significant difference in smoking status was detected between the colorectal cancer patients and healthy volunteers, and the colorectal cancer patients had the high frequency of smoking status compared with healthy volunteers (Table 1). It has been reported that smoking habits affect the metabolite profile of the human body, including the blood [24–26]. In addition, habitual diet also seems to influence the serum metabolite profile [27]. Recently, a positive association between smoking and colorectal cancer was detected in Japanese [28], and so it might be necessary to include information about the lifestyle factors related to colorectal cancer without any lymph node or distant metastasis in predictive models for the disease. In a previous study, it was revealed that the power of a metabolite-based model to predict colorectal cancer was enhanced by incorporating four general clinical factors, age, gender, smoking status, and alcohol status, although the study in question included patients with stage I to IV colorectal cancer [29]. In addition, it was reported that the administration of aspirin and hormonal agents reduced the risk of colorectal cancer [30, 31]. Regarding aspirin, its use decreased the plasma level of oncometabolite 2-hydroxyglutarate in a randomized, double-blind, crossover trial [32], suggesting that medication might cause changes in the plasma levels of certain metabolites. In our study, information about the drugs being taken could not be collected for all subjects in the medication-positive group, so the positive/negative associations between particular medications and plasma metabolite levels should be discussed in the future.

In conclusion, a predictive model for detecting cases of colorectal cancer that do not involve lymph node or distant metastasis was established using a GC/QqQMS based-metabolomics approach. In this study, we focused on stage 0/I/II colorectal cancer, and the diagnostic model was established. Accuracy of this diagnostic model for stage III/IV colorectal cancer has not been investigated, although the previous model [4] was evaluated for all stage (0/I/II/III/IV) colorectal cancer, so the established model may not be appropriate for stage III/IV colorectal cancer. The aim of this study is to establish the diagnostic model to discovery cases of colorectal cancer that do not involve lymph node or distant metastasis, and the metabolites on the model may be different between the previous and the current studies. Our predictive model exhibited high sensitivity and specificity in the present study population, and our results formed the basis of future's prospective studies for other cohorts, so its performance should be prospectively validated in other populations in which blood samples should be collected via the same procedure. In addition, it is also needed to perform the further validation study in general population based on healthy subjects and then to evaluate sensitivity of this model via endoscopy as the gold standard procedure. Specimens from colorectal cancer patients were collected from clinically-diagnosed subjects, so the results might not be representative of those in preclinical cancers involved in healthy population, even though the stages of cancers in the present study were 0 or I. Sensitivity calculated with clinically-diagnosed cancers generally tends to be overestimated than the true sensitivity for preclinical cancers in the population. Our study sample set had the significant differences in the plasma levels of total cholesterol, triglyceride, glucose, total bilirubin and AST between the colorectal cancer patients and healthy volunteers, so it is impossible to completely deny the involvement of these factors’ alterations in the establishment of our model at this time. In the future, the contribution of blood biochemical factors including these factors to the diagnostic model should be also performed via the subset analysis in the validation study with the larger number of human samples. Finally, to enable improvements in QOL via the early detection of colorectal cancer, the practical utility of this predictive model should also be studied. Furthermore, the applicability of our model to screening examinations, for example, health checkups, should be examined.

## MATERIALS AND METHODS

### Subjects

This study was approved by the ethics committees at Kobe University Graduate School of Medicine and the National Cancer Center Japan. We included patients who were diagnosed with stage 0, I or II colorectal cancer at the National Cancer Center Hospital; were histologically confirmed to have adenocarcinoma; and whose blood plasma was collected between May 2011 and July 2014 for research purpose. Patients who were simultaneously diagnosed with another type of cancer or had a history of cancer were excluded. The control plasma samples were obtained from healthy individuals who underwent cancer screening at the Research Center for Cancer Prevention and Screening, National Cancer Center, and whose blood plasma were available for researches. Blood was transferred into tubes containing EDTA-2Na as an anticoagulant. Blood collected from colorectal cancer patients was kept at room temperature for 15-30 min after gentle mixing, and then was kept at 4°C. After 0.5 – 24 hr, the blood was centrifuged at 3,000 rpm for 10 min at 4°C, and plasma was obtained. Blood collected from healthy volunteers was kept at room temperature for about 30 min after gentle mixing, and then was kept at 4°C. After 1 – 6 hr, the blood was centrifuged at 3,000 rpm for 10 min at 4°C, and plasma was obtained. Individuals who were diagnosed with cancer or had previously been diagnosed with cancer or colorectal polyps were excluded from the healthy control group. The colorectal cancer patients were classified into 5-year age groups (40-44, 45-49, 50-54,…, 80-84, 85+), and healthy volunteers were selected by matching them as closely as possible with the colorectal cancer patients based on gender, age group, and the year of blood collection. All plasma samples were prospectively collected from individuals who had provided written informed consent to allow their blood samples to be used for research purposes and were stored at -80°C at the National Cancer Center Biobank (the samples from the colorectal cancer patients) or the Research Center for Cancer Prevention and Screening (the samples from the healthy volunteers). We retrospectively selected 282 colorectal cancer patients and 291 healthy volunteers for our analyses based on the patient/healthy control selection criteria and collected their plasma samples. The subjects’ characteristics are summarized in Table 1. Clinical staging was performed based on the Union for International Cancer Control TNM Classification (7th edition). The classification of histology and tumor location was conducted based on the Japanese Classification of Colorectal Carcinoma (8th edition). In this study, each sample was numbered in a blinded manner before metabolite extraction, and steps until peak identification and alignment were performed by using this blinded number.

### Chemicals and reagents

^13^C_3_-lactic acid, ^13^C_2_-oxalic acid, ^2^H_3_-sacrosine, ^2^H_8_-valine, ^13^C_3_-dihydroxyacetone, ^2^H_10_-isoleucine, ^13^C_4_-fumaric acid, ^13^C_4_-malic acid, ^2^H_3_-aspartic acid, ^13^C_5_-glutamic acid, ^13^C_6_-4-hydroxybenzoic acid, ^2^H_3_-lauric acid, ^13^C_5_-ribose, ^13^C_2_-taurine, ^2^H_4_-citric acid, ^2^H_7_-ornithine, ^13^C_6_-tyrosine, ^13^C_6_-dopa, ^2^H_6_-kynurenine, ^2^H_8_-cystamine, and ^13^C_11_-tryptophan were purchased from Cambridge Isotope Laboratories, Inc. (MA, USA). ^2^H_3_-2-hydorxybutyric acid and 2-isopropylmalic acid were purchased from CDN isotopes (Quebec, CA) and Sigma Aldrich (Tokyo, Japan), respectively. The compounds were dissolved in methanol, as shown in Supplementary Table 3, and then the obtained solution was used as an extraction solution. A standard alkane series mixture (C7 to C33) and octafluoronaphthalene (OFN) were purchased from Restek Co. (PA, USA) and Shimadzu Co. (Kyoto, Japan), respectively. Methoxyamine hydrochloride and N-methyl-N-trimethylsilyl-trifluoroacetamide (MSTFA), which were used for the derivatization, were obtained from Sigma-Aldrich and GL Science (Tokyo, Japan), respectively.

### Sample preparation

To extract low molecular weight metabolites, 50 μL of plasma were mixed with 270 μL of the extraction solution (Supplementary Table 3), and then the mixture was shaken at 1,200 rpm for 30 min at 37°C, before being centrifuged at 2,000 x g for 10 min at 4°C. One hundred μL of the obtained supernatant were transferred to a clean tube and then dried for 3 hr with a centrifugal evaporator. For oximation, 80 μL of 20 mg/ml methoxyamine hydrochloride dissolved in pyridine were added to the tube and then sonicated for 10 min, before being shaken at 1,200 rpm for 90 min at 30°C. The mixture was centrifuged at 2,000 x g for 10 min at 20°C, and then 40 μL of the resultant supernatant were subjected to GC/QqQMS, as described in the next section.

### GC/QqQMS procedure

The GC/QqQMS analysis was performed on an AOC-6000 (Shimadzu Co.) and a GCMS-TQ8040 (Shimadzu Co.) equipped with a BPX-5 capillary column (internal diameter: 30 m × 0.25 mm; film thickness: 0.25 μm; SEG, Victoria, Australia). In the AOC-6000, 20 μL of MSTFA were added to the sample supernatant, and then the mixture was incubated at 750 rpm for 30 min at 37°C, before 1.0 μL of the derivatized solution was injected into the GCMS-TQ8040. During the GCMS-TQ8040 analysis, the inlet temperature was kept at 250°C, and helium was used as a carrier gas at a constant flow rate of 39.0 cm per sec. The injector split ratio was set to 1:30. The GC column temperature was programmed to remain at 60°C for 2 min and then rise from 60°C to 330°C at a rate of 15°C per 1 min, before being kept at 330°C for 3 min. The total GC run time was 23 min. The transfer line and ion-source temperature were 280°C and 200°C, respectively. The ionization voltage was 70 eV. Argon gas was used as a collision-induced dissociation gas. The MRM cycle time was set at 5 cycles/sec to allow accurate peak area assessment. The metabolite detection was performed using the Smart Metabolites Database (Shimadzu, Co.), which contained the relevant MRM method file and data regarding the GC analytical conditions, MRM parameters, and retention index employed for the metabolite measurement. To correct the retention time, the Automatic Adjustment of Retention Time (AART) function of the GCMSsolution software (Shimadzu Co.) and a standard alkane series mixture (C7 to C33) were used. The peak identification was performed automatically and then confirmed manually based on the specific precursor and product ions, and the retention time. The database used in this study includes data about 215 peaks from 153 metabolites, 22 corresponding stable isotopes, and 2-isopropylmalic acid. Due to derivatization-induced variations, 35 metabolites produce 2 peaks each, and 2 metabolites have 3 peaks each. The peak intensity of each metabolite was normalized to that of the internal standard; i.e., 4-hydroxybenzoic acid-^13^C_6_-2TMS. Regarding the metabolites that we identified based on the stable isotopes, their peak intensities were corrected using the corresponding stable isotopes. In this study, 22 stable isotopes were used, but some native metabolites were not detected in the subjects’ plasma. During the normalization process, 22 metabolites were corrected using both the internal standard and the corresponding stable isotopes. Then, the data that exhibited the best co-efficient of variation (CV) during the quality control (QC) process was adopted for the study. Based on the elimination process conducted as part of the QC protocol described in the next section, 64 metabolites were finally selected as reliable targets for statistical evaluation. The MRM parameters for the detected metabolites were shown in Supplementary Table 4. The 2-isopropylmalic acid contained in the extraction solution was also used to evaluate the stability of our GC/QqQMS analysis system.

### Quality controls

In order to ensure the reproducibility of the acquired metabolomic data, a QC procedure is required [33]. We established a specific data cleansing and QC protocol before the analyses. Commercially available pooled plasma (Kohjin-Bio Co., Saitama, Japan) was used as the QC sample. All QC plasma samples were derived from the same product lot. One QC sample was analyzed with each batch of 10 study samples. The QC samples were prepared and analyzed in the same manner as the study samples. An OFN sample (concentration: 100 pg/μL) was also analyzed in each batch of 10 study samples. The criteria used to eliminate samples and metabolites from the study were as follows: 1) samples for which the area ratios of the internal standard; i.e., 4-hydroxybenzoic acid-^13^C_6_-2TMS, were <0.5 or >1.5 times the median value for the batch; 2) samples in which ≥2 stable isotopes that were subjected to metabolite extraction could not be identified; 3) metabolites that could not be detected in ≥1 QC or study samples; 4) metabolites for which the CV for all QC samples was >30% after normalization using the internal standard, or >20% after normalization with the corresponding stable isotopes; and 5) batches in which a signal value of <2,000 is detected in 100 pg/μL of OFN.

### Statistical analysis

Numerical data regarding age, BMI, and the blood test results are presented as the mean and standard deviation (S.D.) for each group, and were compared using the Student's t-test. Categorical data concerning sex, cancer stage, or medical questionnaire responses are presented as distribution charts, and were compared using Pearson's chi-square test. In the comprehensive analysis of metabolites, the plasma levels of each metabolite are presented as mean and S.D. values and were also compared using the Wilcoxon's rank sum test. Ratios of the level of a particular metabolite in the colorectal cancer patients to that seen in the healthy volunteers are shown as fold change values. For clinical variables, p-values of <0.05 were considered to indicate a significant difference. For the metabolite analysis, p-values indicating significant differences were adjusted using the Bonferroni method. The stepwise method was used to select variables for the multivariate analysis. The multicollinearity of the selected variables was assessed by calculating VIF values. The multivariate analysis was performed using multiple logistic regression analysis. Receiver operating characteristic (ROC) curve analysis was used to evaluate the diagnostic performance of the resultant regression model based on its AUC, sensitivity, and specificity values. These analyses were performed using the default conditions of JMP12 (SAS Institute Inc.).

## SUPPLEMENTARY MATERIALS FIGURES AND TABLES






