# Supplementary material for: Investigations in the possibility of early detection of colorectal cancer by gas chromatography/triple-quadrupole mass spectrometry
Source: Oncotarget. 2017 Feb 4;8(10):17115–26. doi: 10.18632/oncotarget.15081 (PMC5370027; doi:10.18632/oncotarget.15081)
Supplement: Supplementary file 1 [file oncotarget-08-17115-s001.pdf]

Investigations in the possibility of early detection of colorectal cancer by gas chromatography/triple-quadrupole mass spectrometry

SUPPLEMENTARY FIGURE AND TABLES

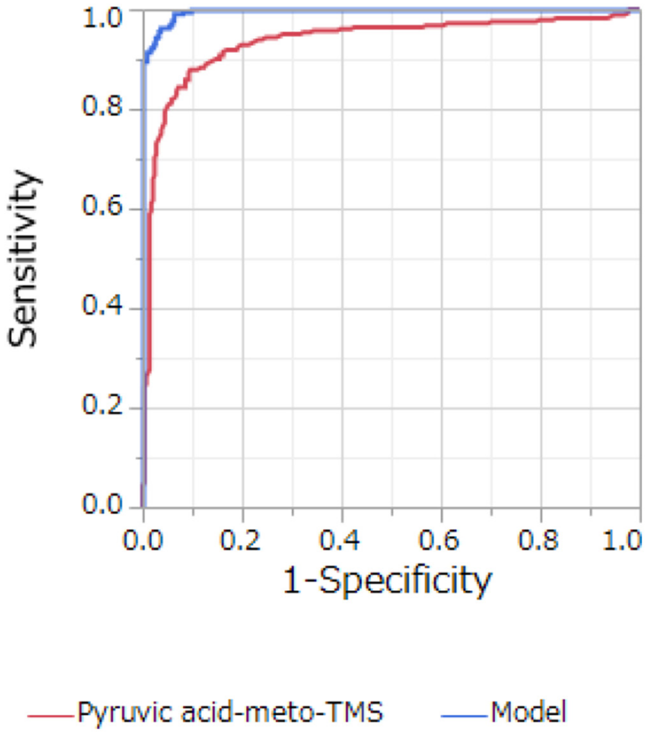

	AUC	S.E.	Lower 95%CI	Upper 95%CI	p-value
Pyruvic acid-meto-TMS	0.936	0.0114	0.909	0.955	<0.0001
Model	0.996	0.00120	0.993	0.998	

Supplementary Figure 1: Comparison between pyruvic acid-meto-TMS and the prediction model. In the ROC curve, the red and blue lines show the results obtained for pyruvic acid-meto-TMS, which was selected via simple linear regression analysis, and the predictive model obtained via multiple logistic regression analysis, respectively. The AUC, S.E., lower 95% confidence interval (CI), upper 95%CI, and p-values for pyruvic acid-meto-TMS and the predictive model are shown in the table below the graph. AUC: area under the curve; S.E.: standard error; CI: confidence interval; TMS: trimethylsilyl group; '-TMS': the number of TMS molecules bound to pyruvic acid via derivatization.

Supplementary Table 1: Blood biochemistry data for the colorectal cancer patients and healthy volunteers

			CRC		HV		p-value
	Units	N	Mean	S.D.	Mean	S.D.	CRC vs. HV
Total cholesterol	mg/dL	219/291	203.1	40.9	213.5	34.3	0.0027
Triglycerides	mg/dL	215/291	122.4	77.9	100.3	54.4	0.0004
HbA1c	%	281/291	5.94	4.13	5.80	0.584	0.561
Glucose	mg/dL	282/291	109.2	24.2	101.9	17.7	<0.0001
Total bilirubin	mg/dL	282/291	0.699	0.262	0.938	0.325	<0.0001
AST	U/L	282/291	22.6	9.52	24.1	7.56	0.0307
ALT	U/L	282/291	21.0	13.6	22.8	10.9	0.0860
BUN	mg/dL	282/291	15.3	4.50	15.5	4.34	0.638
Cre	mg/dL	282/291	0.775	0.227	0.771	0.229	0.854
CRP	mg/dL	276/282	0.247	0.761	0.133	0.238	0.0184
CEA	ng/mL	280/252	5.65	18.4	2.22	1.37	0.0020
CA19-9	U/mL	280/252	16.8	20.8	13.1	10.8	0.0084

The p-values for total cholesterol, triglycerides, HbA1c, glucose, total bilirubin, AST, ALT, CRP, CEA, and CA19-9 were calculated using Welch's t-test. The number of subjects for which data were obtained varied among the parameters, so the mean and S.D. of the collected data were calculated. The numbers of subjects for whom data were collected are shown. The p-values for BUN and Cre were calculated using the Student's t-test. CRC: colorectal cancer patients; HV: healthy volunteers; HbA1c: hemoglobin A1c; AST: aspartate aminotransferase; ALT: alanine aminotransferase; BUN: blood urea nitrogen; Cre: creatinine; CRP: C-reactive protein; CEA: carcinoembryonic antigen; CA19-9: carbohydrate antigen 19-9; S.D.: standard deviation.

Supplementary Table 2: Comparison of plasma metabolite levels between the colorectal cancer patients and healthy volunteers

See Supplementary File 1

Supplementary Table 3: Concentrations of stable isotopes and 2-isopropylmalic acid in the extraction solution

Compound name	Concentration in the extraction solution (µg/mL)	Content in 270 µL of the extraction solution (µg/sample)
$^{13}C_3$-lactic acid	74.07	20.00
$^{2}H_3$-2-hydroxybutyric acid	1.85	0.50
$^{13}C_2$-oxalic acid	1.85	0.50
$^{2}H_3$-sacrosine	1.85	0.50
$^{2}H_8$-valine	18.52	5.00
$^{13}C_3$-dihydroxyacetone	1.85	0.50
$^{2}H_{10}$-isoleucine	1.85	0.50
$^{13}C_4$-fumaric acid	1.85	0.50
$^{13}C_4$-malic acid	1.85	0.50
$^{2}H_3$-aspartic acid	1.85	0.50
$^{13}C_5$-glutamic acid	18.52	5.00
$^{13}C_6$-4-hydroxybenzoic acid	1.85	0.50
$^{2}H_3$-lauric acid	18.52	5.00
$^{13}C_5$-ribose	1.85	0.50
$^{13}C_2$-taurine	74.07	20.00
$^{2}H_4$-citric acid	18.52	5.00
$^{2}H_7$-ornithine	1.85	0.50
$^{13}C_6$-tyrosine	1.85	0.50
$^{13}C_6$-dopa	1.85	0.50
$^{2}H_6$-kynurenine	18.52	5.00
$^{2}H_8$-cystamine	18.52	5.00
$^{13}C_{11}$-tryptophan	1.85	0.50
2-isopropylmalic acid	18.52	5.00

The concentrations of the stable isotopes and 2-isopropylmalic acid dissolved in the extraction solution and their concentrations in 270 µL of the extraction solution (1 sample) are shown.

Supplementary Table 4: MRM parameters for the detected metabolites

See Supplementary File 2
