# Supplementary material for: Investigations in the possibility of early detection of colorectal cancer by gas chromatography/triple-quadrupole mass spectrometry
Source: Oncotarget. 2017 Feb 4;8(10):17115–26. doi: 10.18632/oncotarget.15081 (PMC5370027; doi:10.18632/oncotarget.15081)
Supplement: Supplementary file 2 [file oncotarget-08-17115-s002.docx]

**Supplemental Table 2. Comparison of plasma metabolite levels between the colorectal cancer patients and healthy volunteers**

|  | CRC | |  | HV | |  | Fold change (CRC/HV) |  | p-value (CRC vs. HV) |
| --- | --- | --- | --- | --- | --- | --- | --- | --- | --- |
|  | Mean | S.D. |  | Mean | S.D. |  |  |  |  |
| Pyruvic acid-meto-TMS | 0.502 | 0.198 |  | 0.166 | 0.102 |  | 3.02 |  | <0.0001 |
| Glycolic acid-2TMS | 0.144 | 0.0265 |  | 0.107 | 0.0223 |  | 1.34 |  | <0.0001 |
| Alanine-2TMS | 0.947 | 0.310 |  | 0.863 | 0.251 |  | 1.10 |  | 0.0010 |
| 2-keto-isovaleric acid-meto-TMS | 0.0121 | 0.00324 |  | 0.0127 | 0.00298 |  | 0.951 |  | 0.0130 |
| Glycine-2TMS | 0.0590 | 0.0219 |  | 0.0611 | 0.0219 |  | 0.965 |  | 0.209 |
| Sarcosine-2TMS | 0.113 | 0.0200 |  | 0.114 | 0.0196 |  | 0.993 |  | 0.736 |
| 2-aminoisobutyric acid-2TMS | 0.0361 | 0.00958 |  | 0.0363 | 0.00931 |  | 0.994 |  | 0.980 |
| 3-hydroxybutyric acid-2TMS | 3.11 | 4.03 |  | 2.88 | 2.51 |  | 1.08 |  | 0.0549 |
| 2-aminobutyric acid-2TMS | 0.0244 | 0.00980 |  | 0.0283 | 0.00964 |  | 0.865 |  | <0.0001 |
| 3-hydroxyisovaleric acid-2TMS | 0.106 | 0.0495 |  | 0.109 | 0.0231 |  | 0.965 |  | 0.0001 |
| Urea-2TMS | 24.6 | 6.96 |  | 24.8 | 6.11 |  | 0.989 |  | 0.374 |
| Serine-2TMS | 0.0327 | 0.00969 |  | 0.0338 | 0.00931 |  | 0.967 |  | 0.0981 |
| Leucine-2TMS | 4.79 | 1.63 |  | 5.32 | 1.64 |  | 0.901 |  | 0.0001 |
| Octanoic acid-TMS | 0.208 | 0.0600 |  | 0.203 | 0.0589 |  | 1.02 |  | 0.433 |
| Phosphoric acid-3TMS | 5.86 | 1.22 |  | 6.14 | 1.29 |  | 0.954 |  | 0.0124 |
| Proline-2TMS | 5.11 | 2.50 |  | 4.44 | 2.14 |  | 1.15 |  | 0.0007 |
| Glyceric acid-3TMS | 0.159 | 0.0530 |  | 0.156 | 0.0575 |  | 1.02 |  | 0.133 |
| Nonanoic acid-TMS | 0.813 | 0.204 |  | 0.817 | 0.210 |  | 0.995 |  | 0.898 |
| Threonine-3TMS | 0.650 | 0.275 |  | 0.684 | 0.270 |  | 0.951 |  | 0.115 |
| Threitol-4TMS | 0.00841 | 0.00405 |  | 0.00696 | 0.00293 |  | 1.21 |  | <0.0001 |
| meso-erythritol-4TMS | 0.0464 | 0.251 |  | 0.0336 | 0.0913 |  | 1.38 |  | <0.0001 |
| 4-hydroxyproline-3TMS | 0.197 | 0.116 |  | 0.188 | 0.120 |  | 1.04 |  | 0.180 |
| Cysteine-3TMS | 0.0965 | 0.0418 |  | 0.129 | 0.0648 |  | 0.747 |  | <0.0001 |
| Creatinine-3TMS | 0.0210 | 0.00850 |  | 0.0195 | 0.00781 |  | 1.08 |  | 0.0319 |
| 2-ketoglutaric acid-meto-2TMS | 0.0522 | 0.0162 |  | 0.0438 | 0.0117 |  | 1.19 |  | <0.0001 |
| Phenylalanine-2TMS | 1.18 | 0.396 |  | 1.22 | 0.355 |  | 0.968 |  | 0.179 |
| Xylose-meto-4TMS(2) | 0.00818 | 0.00947 |  | 0.00545 | 0.00279 |  | 1.50 |  | <0.0001 |
| Arabinose-meto-4TMS | 0.0257 | 0.00990 |  | 0.0199 | 0.00674 |  | 1.30 |  | <0.0001 |
| Ribulose-meto-4TMS | 0.0105 | 0.00380 |  | 0.0111 | 0.00342 |  | 0.942 |  | 0.0017 |
| Lauric acid-TMS | 0.0825 | 0.0434 |  | 0.0896 | 0.0436 |  | 0.921 |  | 0.0063 |
| Xylitol-5TMS | 0.0531 | 0.0818 |  | 0.0430 | 0.0540 |  | 1.23 |  | 0.0003 |
| Arabitol-5TMS | 0.0460 | 0.0708 |  | 0.0373 | 0.0475 |  | 1.23 |  | 0.0003 |
| Isocitric acid-4TMS | 0.0412 | 0.0156 |  | 0.0354 | 0.0117 |  | 1.17 |  | <0.0001 |
| 2-aminopimelic acid-3TMS | 0.456 | 0.165 |  | 0.449 | 0.164 |  | 1.02 |  | 0.593 |
| 1,5-anhydro-glucitol-4TMS | 0.0384 | 0.0188 |  | 0.0390 | 0.0173 |  | 0.986 |  | 0.477 |
| Sorbose-meto-5TMS(1) | 0.145 | 0.185 |  | 0.0926 | 0.0668 |  | 1.57 |  | <0.0001 |
| Fructose-meto-5TMS(2) | 0.137 | 0.187 |  | 0.0818 | 0.0600 |  | 1.67 |  | <0.0001 |
| 5-dehydroquinic acid-meto-4TMS | 0.103 | 0.0316 |  | 0.107 | 0.0227 |  | 0.968 |  | 0.0079 |
| Glucose-meto-5TMS(1) | 17.9 | 5.372 |  | 18.5 | 3.872 |  | 0.968 |  | 0.0050 |
| Hippuric acid-TMS | 0.184 | 0.0522 |  | 0.186 | 0.0398 |  | 0.988 |  | 0.133 |
| Galactose-meto-5TMS(2) | 10.9 | 3.37 |  | 11.1 | 2.732 |  | 0.978 |  | 0.0858 |
| Glucosamine-5TMS(1) | 0.182 | 0.062 |  | 0.187 | 0.0487 |  | 0.973 |  | 0.0341 |
| Lysine-4TMS | 0.970 | 0.299 |  | 1.10 | 0.276 |  | 0.884 |  | <0.0001 |
| Glucuronic acid-meto-5TMS(1) | 0.0585 | 0.0420 |  | 0.0560 | 0.0214 |  | 1.04 |  | 0.165 |
| Ascorbic acid-4TMS | 0.00779 | 0.00283 |  | 0.00807 | 0.00216 |  | 0.966 |  | 0.0238 |
| Glucaric acid-6TMS | 0.00627 | 0.00310 |  | 0.00672 | 0.00380 |  | 0.932 |  | 0.0085 |
| Palmitoleic acid-TMS | 0.0262 | 0.0187 |  | 0.0362 | 0.0222 |  | 0.722 |  | <0.0001 |
| Inositol-6TMS | 0.788 | 0.276 |  | 0.757 | 0.232 |  | 1.04 |  | 0.401 |
| Uric acid-4TMS | 1.30 | 0.639 |  | 1.53 | 0.672 |  | 0.850 |  | <0.0001 |
| Kynurenine-3TMS | 0.00652 | 0.00308 |  | 0.00649 | 0.00231 |  | 1.00 |  | 0.341 |
| Elaidic acid-TMS | 0.138 | 0.0594 |  | 0.156 | 0.0532 |  | 0.885 |  | <0.0001 |
| Sucrose-8TMS | 0.0358 | 0.0680 |  | 0.0234 | 0.228 |  | 1.53 |  | <0.0001 |
| Maltose-meto-8TMS(1) | 0.00585 | 0.00741 |  | 0.00546 | 0.00325 |  | 1.07 |  | <0.0001 |
| Lactic acid-2TMS(/SI) | 0.256 | 0.0720 |  | 0.162 | 0.0552 |  | 1.59 |  | <0.0001 |
| 2-hydroxybutyric acid-2TMS(/SI) | 0.683 | 0.342 |  | 0.675 | 0.236 |  | 1.01 |  | 0.433 |
| Valine-2TMS(/SI) | 2.64 | 0.609 |  | 2.82 | 0.576 |  | 0.933 |  | <0.0001 |
| Isoleucine-2TMS(/SI) | 1.04 | 0.273 |  | 1.02 | 0.268 |  | 1.01 |  | 0.360 |
| Fumaric acid-2TMS(/SI) | 0.0185 | 0.00504 |  | 0.0132 | 0.00400 |  | 1.40 |  | <0.0001 |
| Malic acid-3TMS(/SI) | 0.0425 | 0.0189 |  | 0.0351 | 0.0120 |  | 1.21 |  | <0.0001 |
| Glutamic acid-3TMS(/SI) | 0.114 | 0.0498 |  | 0.120 | 0.0520 |  | 0.949 |  | 0.139 |
| Citric acid-4TMS(/SI) | 0.262 | 0.0652 |  | 0.260 | 0.0588 |  | 1.01 |  | 0.817 |
| Ornithine-4TMS(/SI) | 0.783 | 0.265 |  | 0.572 | 0.176 |  | 1.37 |  | <0.0001 |
| Tyrosine-3TMS(/SI) | 1.04 | 0.254 |  | 1.09 | 0.212 |  | 0.959 |  | 0.0034 |
| Tryptophan-3TMS(/SI) | 1.03 | 0.227 |  | 1.15 | 0.209 |  | 0.895 |  | <0.0001 |

The p-values were calculated using Wilcoxon’s rank sum test. CRC: colorectal cancer patients; HV: healthy volunteers; S.D.: standard deviation; TMS: trimethylsilyl group; SI: stable isotope; ‘-TMS’: the number of TMS molecules bound to each metabolite via derivatization; ‘/SI’: the metabolites whose peak intensity values were normalized using the corresponding stable isotopes
