# Supplementary material for: Investigations in the possibility of early detection of colorectal cancer by gas chromatography/triple-quadrupole mass spectrometry
Source: Oncotarget. 2017 Feb 4;8(10):17115–26. doi: 10.18632/oncotarget.15081 (PMC5370027; doi:10.18632/oncotarget.15081)
Supplement: Supplementary file 3 [file oncotarget-08-17115-s003.docx]

**Supplemental Table 4. MRM parameters for the detected metabolites**

|  |  |  |  | Target ion | |
| --- | --- | --- | --- | --- | --- |
|  | Retention index | Retention time (min) |  | Transition | Collision energy (eV) |
| Pyruvic acid-meto-TMS | 1046 | 6.270 |  | 174.00-74.00 | 21 |
| Glycolic acid-2TMS | 1069 | 6.545 |  | 177.10-147.10 | 6 |
| Alanine-2TMS | 1095 | 6.856 |  | 190.10-147.10 | 9 |
| 2-keto-isovaleric acid-meto-TMS | 1102 | 6.938 |  | 186.10-73.00 | 18 |
| Glycine-2TMS | 1116 | 7.091 |  | 176.10-147.10 | 12 |
| Sarcosine-2TMS | 1131 | 7.255 |  | 116.10-73.00 | 9 |
| 2-aminoisobutyric acid-2TMS | 1135 | 7.299 |  | 130.10-114.10 | 6 |
| 3-hydroxybutyric acid-2TMS | 1152 | 7.485 |  | 191.10-147.10 | 12 |
| 2-aminobutyric acid-2TMS | 1164 | 7.616 |  | 204.20-147.10 | 6 |
| 3-hydroxyisovaleric acid-2TMS | 1200 | 8.010 |  | 131.10-73.00 | 12 |
| Urea-2TMS | 1239 | 8.404 |  | 171.10-73.00 | 18 |
| Serine-2TMS | 1250 | 8.515 |  | 234.10-73.00 | 21 |
| Leucine-2TMS | 1260 | 8.616 |  | 158.10-73.00 | 15 |
| Octanoic acid-TMS | 1260 | 8.616 |  | 201.10-75.00 | 18 |
| Phosphoric acid-3TMS | 1260 | 8.616 |  | 314.10-73.00 | 24 |
| Proline-2TMS | 1291 | 8.929 |  | 142.10-73.00 | 18 |
| Glyceric acid-3TMS | 1312 | 9.132 |  | 189.10-73.00 | 18 |
| Nonanoic acid-TMS | 1357 | 9.555 |  | 215.10-75.00 | 18 |
| Threonine-3TMS | 1367 | 9.649 |  | 218.10-73.00 | 18 |
| Threitol-4TMS | 1471 | 10.584 |  | 307.10-73.00 | 24 |
| meso-erythritol-4TMS | 1480 | 10.664 |  | 307.00-217.10 | 6 |
| 4-hydroxyproline-3TMS | 1511 | 10.931 |  | 230.10-73.00 | 24 |
| Cysteine-3TMS | 1543 | 11.196 |  | 220.10-73.00 | 24 |
| Creatinine-3TMS | 1548 | 11.237 |  | 329.20-115.10 | 21 |
| 2-ketoglutaric acid-meto-2TMS | 1566 | 11.386 |  | 198.10-73.00 | 15 |
| Phenylalanine-2TMS | 1623 | 11.847 |  | 218.10-73.00 | 15 |
| Xylose-meto-4TMS(2) | 1626 | 11.870 |  | 307.10-103.10 | 15 |
| Arabinose-meto-4TMS | 1632 | 11.917 |  | 307.10-73.10 | 24 |
| Ribulose-meto-4TMS | 1646 | 12.027 |  | 263.10-173.10 | 9 |
| Lauric acid-TMS | 1647 | 12.035 |  | 257.10-75.00 | 18 |
| Xylitol-5TMS | 1672 | 12.231 |  | 307.10-73.00 | 24 |
| Arabitol-5TMS | 1685 | 12.332 |  | 307.10-217.10 | 6 |
| Isocitric acid-4TMS | 1793 | 13.140 |  | 245.10-73.00 | 21 |
| 2-aminopimelic acid-3TMS | 1794 | 13.147 |  | 274.20-73.00 | 24 |
| 1,5-anhydro-glucitol-4TMS | 1831 | 13.410 |  | 362.20-257.10 | 12 |
| Sorbose-meto-5TMS(1) | 1833 | 13.424 |  | 307.10-73.00 | 21 |
| Fructose-meto-5TMS(2) | 1844 | 13.502 |  | 307.10-217.20 | 6 |
| 5-dehydroquinic acid-meto-4TMS | 1858 | 13.601 |  | 300.10-210.10 | 9 |
| Glucose-meto-5TMS(1) | 1861 | 13.622 |  | 160.10-105.10 | 6 |
| Hippuric acid-TMS | 1862 | 13.629 |  | 206.10-190.10 | 12 |
| Galactose-meto-5TMS(2) | 1879 | 13.749 |  | 205.10-117.10 | 9 |
| Glucosamine-5TMS(1) | 1884 | 13.784 |  | 203.10-73.00 | 18 |
| Lysine-4TMS | 1900 | 13.897 |  | 317.20-156.20 | 9 |
| Glucuronic acid-meto-5TMS(1) | 1902 | 13.911 |  | 333.10-73.00 | 27 |
| Ascorbic acid-4TMS | 1926 | 14.072 |  | 332.10-215.10 | 18 |
| Glucaric acid-6TMS | 1977 | 14.416 |  | 333.10-73.00 | 24 |
| Palmitoleic acid-TMS | 2026 | 14.738 |  | 311.10-131.10 | 12 |
| Inositol-6TMS | 2058 | 14.943 |  | 305.10-73.00 | 21 |
| Uric acid-4TMS | 2082 | 15.097 |  | 456.20-441.10 | 18 |
| Kynurenine-3TMS | 2186 | 15.740 |  | 307.10-218.10 | 9 |
| Elaidic acid-TMS | 2221 | 15.949 |  | 339.20-75.00 | 18 |
| Sucrose-8TMS | 2579 | 17.927 |  | 361.10-73.00 | 24 |
| Maltose-meto-8TMS(1) | 2678 | 18.428 |  | 361.10-169.10 | 15 |
| Lactic acid-2TMS | 1051 | 6.330 |  | 219.00-147.10 | 15 |
| 2-hydroxybutyric acid-2TMS | 1117 | 7.102 |  | 205.10-147.10 | 9 |
| Valine-2TMS | 1204 | 8.051 |  | 218.10-147.10 | 9 |
| Isoleucine-2TMS | 1281 | 8.828 |  | 158.20-73.00 | 12 |
| Fumaric acid-2TMS | 1344 | 9.433 |  | 245.00-147.10 | 15 |
| Malic acid-3TMS | 1472 | 10.593 |  | 335.00-147.10 | 15 |
| Glutamic acid-3TMS | 1603 | 11.690 |  | 246.10-73.00 | 24 |
| Citric acid-4TMS | 1794 | 13.147 |  | 347.10-147.10 | 24 |
| Ornithine-4TMS | 1796 | 13.162 |  | 142.10-73.00 | 15 |
| Tyrosine-3TMS | 1929 | 14.093 |  | 280.10-73.00 | 18 |
| Tryptophan-3TMS | 2214 | 15.908 |  | 202.10-73.00 | 18 |

TMS: trimethylsilyl group; ‘-TMS’: the number of TMS molecules bound to each metabolite via derivatization
